# Efficacy of the PEN-FAST score in a French cohort of patients with reported allergy to penicillins

**DOI:** 10.3389/falgy.2024.1439698

**Published:** 2024-07-29

**Authors:** Anatole Hanniet, Marc Puyraveau, Florence Castelain, Fabien Pelletier, François Aubin

**Affiliations:** ^1^ Allergology Unit, Departement of Dermatology, University Hospital, Besançon, France; ^2^ Methodology and Statistics Unit, University Hospital, Besançon, France; ^3^ INSERM UMR 1098 RIGHT, University of Besançon, Besançon, France

**Keywords:** allergy, hypersensitivity, penicillin, PEN-FAST, delabeling

## Abstract

**Introduction:**

Various clinical decision-making tools for penicillin allergy have been developed to guide delabeling strategies.

**Objective:**

To evaluate the penicillin allergy PEN-FAST decision score in a retrospective cohort of patients, adults and children, with penicillin-reported allergy.

**Methods:**

This monocentric retrospective cohort included patients with penicillin-reported allergy. All patients underwent penicillin allergy testing using skin tests and/or drug challenge. The PEN-FAST score sensitivity, specificity, negative (NPV) and positive (PPV) predictive values, and the area under the receiver operating characteristics curve (AUC) were calculated.

**Results:**

Two hundred and fourteen patients were included (64 children and 150 adults). Allergy was confirmed in 52 cases (24%). A PEN-FAST score <3 points showed a poor discrimination capacity for the whole population (AUC = 0.66; 95% CI: 0.58–0.75), while it demonstrated a better discrimination capacity in the adults group (AUC = 0.71; 95% CI: 0.63–0.80). The sensitivity to identify penicillin allergy using this cutoff of less than 3 points was 0.67 (95% CI: 0.52–0.80); specificity, 0.58 (95% CI: 0.48–0.68); PPV, 0.43 (95% CI: 0.32–0.55); and NPV, 0.78 (95% CI: 0.68–0.87).

**Conclusions:**

Although our data confirm a rather good discrimination value of a PEN-FAST score <3 points, its low negative predictive value (78%) did not advocate for its use as an accurate, simple and cost-effective clinical decision-making tool to effectively reduce the number of penicillin skin tests required before direct oral challenge. Further studies are required to improve the predictive capacity of the PEN-FAST score.

## 1 Introduction

Penicillin is the most common patient-reported drug allergy, with a prevalence ranging from 6 to 25% across various regions and treatment populations (1–3). Furthermore, most diagnoses of penicillin allergy are indeed made in childhood when allergic symptoms can be confused with symptoms of viral or bacterial illness. These unverified penicillin allergy labels have been associated with the use of broad-spectrum antibiotics and adverse effects including *C. difficile* infection and antibiotic resistance. The current standard of care in adults to verify or disprove a penicillin allergy includes prick, intradermal and patch skin testing, followed, if negative, by drug challenge. However, after formal allergy assessment with skin testing and drug challenge, less than 5% of persons with a remote and low-risk history of penicillin allergy are found to be truly allergic (1). Given the high prevalence of drug allergy labels and low rate of drug hypersensitivity confirmation, it is essential to identify patients with low-risk penicillin allergy. There are multiple barriers to penicillin allergy delabeling including the fear of inducing an allergic reaction, heavy workload, time-consuming procedures and the shortage of nursing staff and allergists (4). Although different clinical decision-making tools have been developed to identify low-risk patients, there has yet to be a global consensus, and approaches continue to vary between allergy centers (5). A PEN-FAST score <3 points is a clinical decision-making tool with a high negative predictive value (NPV) that can identify patients with low-risk penicillin allergy who could benefit, potentially, from penicillin challenge without prior skin testing (6).

The aim of our study was to evaluate the decision score PEN-FAST <3 points in a retrospective cohort of patients, adults and children, with penicillin-reported allergy.

## 2 Method

### 2.1 Patients

A single-center, retrospective cohort study was conducted on consecutive patients reporting drug hypersensitivity reactions (immediate, delayed, or unknown) to at least one penicillin. This study was approved by the institutional board of Besançon University Hospital (France) under reference 2022/685. All data were already collected prior to inclusion and informed consent was not required. All patients had been tested in the Allergy Unit of Besançon University Hospital between July 1st, 2020 and December 31st, 2021. Data collection was performed between March 25th, 2023 and July 11th, 2023.

### 2.2 Skin tests

Skin tests (pricks, intradermals, patchs) were performed in all patients with the culprit penicillin when known or amoxicillin/clavulanate when unknown following the current European Academy of Allergy and Clinical Immunology (EAACI) (7).

### 2.3 Drug challenges

All patients reporting penicillin hypersensitivity reactions (HSR) and having negative skin tests underwent drug challenge. The target dose for drug challenge was the maximum single therapeutic dose. As recommended in adults (8), we used a two-step gradual challenge including a 10% starting dose followed by 100% of the dose at 30 min interval. For children, we used a one-step drug challenge consisting of 100% maximal single therapeutic dose. After the last dose, patients underwent a 2-hour observation period before being discharged. Patients were invited to contact the Allergology unit at day 7 to declare late reactions. The challenge was considered positive if objective symptoms of immediate hypersensitivity were triggered before the end of the 2-hour observation period or if symptoms of delayed hypersensitivity were observed during the week following the challenge.

### 2.4 PEN-FAST decision rule

The PEN-FAST score includes four clinical variables predictive of a positive penicillin allergy test result (6): HSR within preceding five years or less (F, 2 points); angioedema/anaphylaxis (A) or Severe Cutaneous Adverse Reaction (SCAR) (A and S, 2 points); and treatment required or unknown (T, 1 point). A cut-off value of <3 points was used to define a low risk penicillin allergy (6).

### 2.5 Statistical analysis

The PEN-FAST scores were compared with outcomes based on positive penicillin allergy test results, which were defined as positive skin test results or drug challenge. Sensitivity, specificity, NPV, positive predictive value (PPV) and the area under the receiver operating characteristic curve was calculated to assess overall diagnostic performance. Data were analyzed using Stata version 16.1 (StataCorp).

## 3 Results

### 3.1 Demographic data

Two hundred and fourteen patients (150 adults and 64 children) were analysed. In children, the median age was 6 (IQR 1–11) and the sex ratio was balanced. In adults, the median age was 55 (IQR 38–67) and 66% of patients were female (Table 1).

**Table 1:** Demographic characteristics and description of patient's reactions.

Patients	All *n* = 214 (%)	Children *n* = 64 (%)	Adults *n* = 150 (%)
Age (median, years)	42 (IQR 14–62)	6 (IQR 1–11)	55 (IQR 38–67)
Female	133 (62%)	33 (51%)	100 (66%)
Reactions	All *n* = 216 (%)	Children *n* = 64 (%)	Adults *n* = 152 (%)
Suspected penicillin			
Amoxicillin	123 (57%)	51 (80%)	72 (47%)
Amoxicillin/Clavulanate	46 (21%)	11 (17%)	35 (23%)
Unknown	33 (15%)	0 (0%)	33 (22%)
Piperacillin	12 (5.5%)	2 (3%)	10 (7%)
Bacampicillin	1 (0.5%)	0 (0%)	1 (0.7%)
Cloxacillin	1 (0.5%)	0 (0%)	1 (0.7%)
Associated non-penicillin suspected drug	41 (19%)	8 (12.5%)	33 (22%)
Chronological phenotype			
Immediate	49 (23%)	7 (11%)	42 (28%)
Delayed	56 (26%)	19 (30%)	37 (24%)
Undetermined	109 (51%)	38 (59%)	72 (48%)
Anaphylaxis (ring)	28 (13%)	1 (2%)	27 (18%)
Grade II	17 (8%)	0 (0)	17 (11%)
Grade III	6 (3%)	0 (0)	6 (4%)
Grade IV	0 (0)	0 (0)	0 (0)
Unknown	5 (2%)	1 (2%)	4 (3%)
SCAR	9 (4.2%)	0 (0%)	9 (6%)
AGEP	9 (4.2%)	0 (0%)	9 (6%)
DRESS	0 (0%)	0 (0%)	0 (0%)
TEN/SJS	0 (0%)	0 (0%)	0 (0%)
Reaction within the last 5 years	130 (60%)	55 (86%)	75 (49%)
Treatment			
Yes	77 (36%)	23 (36%)	54 (36%)
No	30 (14%)	11 (17%)	19 (12%)
Unknown	109 (50%)	30 (47%)	79 (52%)
Positive allergy tests	52 (24%)	3 (5%)	49 (32%)
Skin-test	47 (22%)	2 (3%)	45 (30%)
Drug challenge	5 (2%)	1 (2%)	4 (2%)
PEN-FAST≥3 points	120 (56%)	44 (69%)	76 (50%)
Allergy^a^ (true positive)	35 (30%)	2 (5%)	33 (43%)
No allergy (false negative)	85 (70%)	42 (95%)	43 (57%)
PEN-FAST<3 points	96 (44%)	20 (31%)	76 (50%)
Allergy^a^ (false negative)	17 (18%)	1 (5%)	16 (21%)
No allergy (true negative)	79 (82%)	19 (95%)	60 (79%)

SCAR, severe cutaneous adverse reaction; AGEP, acute generalized exanthematous pustulosis; DRESS, drug reaction with eosinophilia and systemic symptoms; TEN/SJS, toxic epidermal necrolysis/ Stevens Johnson syndrome. IQR, interquartile range.

^a^ Positive penicillin allergy tests were defined as positive skin test or drug challenge.

The most frequently suspected drug was amoxicillin, either alone (57%) or associated with clavulanate potassium (21%), both in adults (47% and 23%, respectively) and children (80% and 17%, respectively). In our series, 41 (19%) patients reported an associated non-penicillin drug that could be suspected of triggering the reaction (Table 1). Among these 41 patients, 19 were only positive for penicillin allergy and 2 were positive for both penicillin and the other drug (ceftriaxone and acetylcystein).

The delay between drug exposure and reaction was less than one hour (immediate) in 49 reactions (23%), more than 6 h (delayed) in 56 reactions (26%), and between 1 and 6 h or undetermined in 109 reactions (51%). Most reactions occurred within the last 5 years in child cases (86%), and approximately in half of adult cases (49%).

Severe cutaneous adverse reactions and anaphylaxis were reported by 9 patients and 28 patients respectively. Except for one case of grade II anaphylaxis in a child, all severe reactions were reported among adults.

### 3.2 Skin tests and drug challenge

Allergy was confirmed in 52 patients (24%) either by skin tests in 47/216 cases (22%) or by drug challenge in 5 cases (2%). Allergy was more frequently confirmed in adults (32%) than in children (5%). No adverse reactions were triggered during skin tests, and drug challenges induced 2 grade II anaphylactic reactions. One case displayed lower respiratory symptoms and was treated with salbutamol nebulization, leading to rapid improvement. The other one displayed lower respiratory symptoms associated with abdominal pain and conjunctivitis requiring intramuscular epinephrine injection.

### 3.3 PEN-FAST score performance

Among the 52 patients with positive allergy tests, 35 (67%) were correctly identified by PEN-FAST ≥3 points. However, 17 (33%) patients were misclassified using a PEN-FAST score <3 points.

Among the adults, 16/76 (21%) demonstrated both a negative PEN-FAST score <3 points and positive allergy tests. These patients were considered as allergic based on positive skin tests (14 patients) or after a positive drug challenge (2 patients). The PEN-FAST score <3 points performances (Table 2) demonstrated a 0.67 sensitivity (95% CI: 0.52–0.80), a 0.58 specificity (95% CI: 0.48–0.68), a PPV of 0.43 (95% CI: 0.32–0.55) and a NPV of 0.78 (95% CI: 0.68–0.87). In contrast, among the children (20), only 1 (5%) demonstrated a false negative PEN-FAST score <3 and positive allergy tests. The PEN-FAST score <3 points was associated with a sensitivity (0.67), specificity (0.31), PPV (0.05), NPV (0.95). Furthermore, the PEN-FAST score <3 points showed a poor discrimination capacity for the whole population (AUC = 0.66; 95% CI: 0.58–0.75), while it demonstrated a better discrimination capacity in the adults group (AUC = 0.71; 95% CI: 0.63–0.80) as compared to the children group (AUC = 0.45; 95% CI: 0.07–0.84).

**Table 2:** PEN-FAST performance in adults at different thresholds.

Score value	FN	Se	Sp	PPV	NPV
≥1 *n* = 145/152	0	1 [0,93–1]	0,07 [0,03–0,14]	0,34 [0,26–0,42]	1 [0,59–1]
≥2 *n* = 87/152	10	0,80 [0,66–0,90]	0,53 [0,43–0,63]	0,45 [0,34–0,56]	0,85 [0,74–0,92]
**≥3 *n* = 76/152**	**16**	**0,67** [0,52–0,80]	**0,58** [0,48–0,68]	**0,43** [0,32–0,55]	**0,79** [0,68–0,87]
≥4 *n* = 26/152	31	0,37 [0,23–0,52]	0,92 [0,85–0,97]	0,69 [0,48–0,86]	0,75 [0,67–0,83]

FN, false negative; Se, sensitivity; Sp, specificity; PPV, positive predictive value; NPV, negative predictive value, All estimations are provided with 95% confidence intervals.

Bold values correspond to the threshold proposed by Trubiano et al. (6).

We did not observe any significant difference whatever the severity or the chronological phenotype (immediate vs. delayed) of the penicillin allergy reported reaction. Different thresholds of PEN-FAST scores were evaluated. The best threshold to identify patients with low-risk penicillin allergy who may not require any skin tests was a PEN-FAST score <2 which was associated with a high NPV (Table 2).

## 4 Discussion

The PEN-FAST tool was previously externally validated in a mixed prospective derivation and retrospective validation cohort of patients tested for penicillin allergy from Australia and the US and in 2 additional adult populations (6, 9, 10).

A recent randomized clinical trial demonstrated that direct penicillin challenge in patients with a low-risk penicillin allergy (PEN-FAST <3) was non inferior compared with standard-of-care skin testing followed by drug challenge. However, it is important to note that this analysis was limited to patients with immediate HS after drug challenge (11). A cut-off of less than 3 points for PEN-FAST has been reported as a low risk of penicillin allergy with a negative predictive value ranging from 85% to 100% (Table 3).

**Table 3:** Diagnosis performance of PEN-FAST score <3 in adult reported-penicillin hypersensitivity reactions.

	IHSR	Positive penicillin allergy tests	AUC^d^	Sensitivity	Specificity	PPV^e^	NPV^f^
Trubiano et al. (6) *n* = 622	43%	58 (9.3%)	0,75 [0,68–0,81]	0,71 [0,57–0,82]	0,78 [0,75–0,82]	0,25 [0,19–0,33]	0,96 [0,94–0,98]
Trubiano et al. (6)^a^ *n* = 334	39%	48 (15%)	0.73 [0.66–0.81]	0.87 [0.75–0.95]	0.40 [0.34–0.46]	0.20 [0.14–0.26]	0.95 [0.89-.0.98]
Trubiano et al. (6)^b^ *n* = 80	21%	27 (34%)	0,78 [0,68–0,88]	0,70 [0,50–0,86]	0,85 [0,72–0,93]	0,70 [0,50–0,86]	0,85 [0,72–0,93]
Trubiano et al. (6)^c^ *n* = 531	21%	19 (4%)	0,74 [0,62–0,86]	0,74 [0,05–0,91]	0,60 [0,55–0,64]	0,06 [0,03–0,10]	0,98 [0,96–0,99]
Piotin et al. (9) *n* = 142	100%	94 (66%)	0,86 [0,79–0,92]	0,56 [0,41–0,71]	0,98 [0,93–1]	0,81 [0,73–0,88]	0,93 [0,77–0,99]
Castagna et al. (12) *n* = 252	24%	27 (11%)	0.72 [UKN]	0.65 [UKN]	0.68 [UKN]	0.23 [UKN]	0.93 [UKN]
Su et al. (10) *n* = 120	UKN	4 (3.4%)	0.88 [0.84–0.92]	1.00 [0.40–1.00]	0.76 [0.67–0.83]	0.12 [0.03–0.29]	1.00 [0.96–1.00]
Hanniet et al. (2024) *n* = 152	28%	49 (32%)	0,71 [0,63–0,80]	0,67 [0,52–0,80]	0,58 [0,48–0,68]	0,43 [0,32–0,55]	0,79 [0,68–0,87]

^a^ Perth cohort.

^b^ Sydney cohort.

^c^ Nashville cohort.

^d^ AUC, area under the curve.

^e^ PVV, positive predictive value.

^f^ NPV, negative predictive value; IHSR, immediate hypersensitivity reaction; UKN, unknown.

As expected, only 5% of children with penicillin-reported HSR were confirmed by allergy tests despite a high frequency (69%) of PEN-FAST score ≥3 points. The PEN-FAST score showed a poor discrimination capacity along with a high NPV (95%) and a poor PPV (5%, data not shown). As previoulsy demonstrated in a pediatric population (13), a high NPV of 95% was considered poor in the context of a low prevalence positive challenge (5%). Our data are similar in children with a poor discrimination capacity (AUC 0.45). These negative data may be explained by the fact that the majority of penicillin allergy cases in pediatric patients are self-reported or parent-reported and often inconsistent with a true allergy along with the increased prevalence of viral-induced reactions. Furthermore, the majority of children who report penicillin allergy can tolerate the medication without adverse reactions and testing for allergy (11). There is mounting evidence to support the safety and cost-effectiveness of a direct oral challenge approach in children with low-risk symptoms of allergy (8, 13). Safety and cost effectiveness of supervised ambulatory drug challenge starting with a therapeutic dose of drug during consultation have been previoulsy demonstrated in children (14).

In contrast, when considering adults, 67% with positive skin tests were correctly identified by PEN-FAST ≥3 points as compared to 56% of patients in a prospective cohort of 252 adults patients (12). However, 16 patients (21%) were misclassified in our study using a PEN-FAST score <3 points as compared to 44% of patients in the previous cohort (12). Although our data confirmed the good discrimination value (71%) of a PEN-FAST score <3, its negative predictive value in adults remained low (78%).

We do not have any clear explanation for the data discrepancies in adults but we suggest that the selection of patients included in the studies might be involved. The prevalence of positive allergy tests (skin test and/or drug challenge) was indeed very different, ranging from 3.4% to 66% (Table 3) (6, 9, 10, 12). The prevalence of severe reactions was also different across studies, our series included 28 patients with anaphylaxis (13%, 1 children) and 9 with SCAR (4.2%, no children). However, as shown in the Table 3, despite these disparities, the data of PEN-FAST score <3 were still closed in terms of AUC, Sensitivity, Specificity. Altogether, these data do not suggest that the performances of the PEN-FAST score are impacted by the proportion of IHSR. Furthermore, it has been demonstrated that additional criteria could improve the discrimination capacity of the PEN-FAST score. A recent study (12) identified two potential additional criteria: skin rash lasting more than 7 days and immediate reaction occurring in less than 1 h (generalized or localized on palmoplantar area or scalp itching/heat feeling). However, although the AUC significantly increased with these additional criteria, the NPV of a PEN-FAST score <3 remained similar (about 92%) likely because of memory bias included in the score.

Some limitations of our study could be linked to its retrospective design, referral bias, and single study site.

In conclusion, in contrast to previous studies (6, 9, 10, 12), our real-life study could not promote the use of a PEN-FAST score <3 as an accurate, simple and cost-effective clinical decision-making tool to effectively identify patients with low-risk penicillin allergy. Further studies are required to improve the predictive capacity of the PEN-FAST score. Lowering the threshold at 2 points increases the performance of the score.

## Data Availability

The original contributions presented in the study are included in the article, further inquiries can be directed to the corresponding author.
